# Amphibians of Serra Bonita, southern Bahia: a new hotpoint within Brazil’s Atlantic Forest hotspot

**DOI:** 10.3897/zookeys.449.7494

**Published:** 2014-10-22

**Authors:** Iuri Ribeiro Dias, Tadeu Teixeira Medeiros, Marcos Ferreira Vila Nova, Mirco Solé

**Affiliations:** 1 Departamento de Ciências Biológicas, Universidade Estadual de Santa Cruz, Rodovia Jorge Amado, km, 16, 45662-900 Ilhéus, Bahia, Brasil; 2 Graduate Program in Applied Zoology, Universidade Estadual de Santa Cruz, Rodovia Jorge Amado, km 16, 45662-900 Ilhéus, Bahia, Brasil; 3 Conselho de Curadores das Coleções Científicas, Universidade Estadual de Santa Cruz, Rodovia Jorge Amado, km 16, 45662-900 Ilhéus, Bahia, Brasil

**Keywords:** Anura, Mountain, Biodiversity, species distribution, species richness

## Abstract

We studied the amphibian community of the Private Reserve of Natural Heritage (RPPN) Serra Bonita, an area of 20 km^2^ with steep altitudinal gradients (200–950 m a.s.l.) located in the municipalities of Camacan and Pau-Brasil, southern Bahia State, Brazil. Data were obtained at 38 sampling sites (including ponds and transects within the forest and in streams), through active and visual and acoustic searches, pitfall traps, and opportunistic encounters. We recorded 80 amphibian species distributed in 15 families: Aromobatidae (1), Brachycephalidae (3), Bufonidae (4), Centrolenidae (2), Ceratophryidae (1), Craugastoridae (7), Eleutherodactylidae (2), Hemiphractidae (2), Hylidae (42), Hylodidae (1), Leptodactylidae (7), Microhylidae (3), Siphonopidae (1), Odontophrynidae (3) and Pipidae (1). Species richness was positively correlated with monthly rainfall. Near 36% of the species were found in strictly forest environments, 15% are endemic to Bahia State and 77.2% are endemic to the Atlantic Forest biome. The large species diversity of this small area, the high degree of endemism and the taxonomic and biogeographic significance turn the Serra Bonita mountain into a hotpoint for amphibians within Brazil’s Atlantic Forest hotspot.

## Abstract (pt)

Neste trabalho, estudamos a comunidade de anfíbios da Reserva Particular do Patrimônio Natural (RPPN) Serra Bonita que possui área de 20 km² com gradientes altitudinais abruptos (200–950 m acima do nível do mar), localizada nos municípios de Camacan e Pau Brasil, sul do Estado da Bahia, Brasil. Os dados foram obtidos em 38 diferentes sítios amostrais (incluindo poças, transectos no interior da floresta e em riachos) através de procura ativa visual e acústica, armadilhas de interceptação e queda e por encontros oportunísticos. Foram registradas 80 espécies de anfíbios distribuídas em 15 famílias: Aromobatidae (1), Brachycephalidae (3), Bufonidae (4), Centrolenidae (2), Ceratophryidae (1), Craugastoridae (7), Eleutherodactylidae (2), Hemiphractidae (2), Hylidae (42), Hylodidae (1), Leptodactylidae (7), Microhylidae (3), Siphonopidae (1), Odontophrynidae (3) e Pipidae (1). A riqueza foi positivamente correlacionada com a precipitação mensal. Aproximadamente 36% das espécies foram encontradas em ambientes estritamente florestais, 15% são endêmicas do Estado da Bahia e 77.2% são endêmicas do bioma Mata Atlântica. A alta diversidade de espécies desta pequena área, o elevado endemismo e a relevância taxonômica e biogeográfica tornam a Serra Bonita um ‘hotpoint’ para anfíbios no ‘hotspot’ da Mata Atlântica.

## Introduction

Among vertebrates, amphibians are considered the most threatened group on the planet (Hoffmann et al. 2010). Near a third of the world’s amphibian species are endangered (Stuart et al. 2004). The main threats to the group are loss and fragmentation of habitat (Young et al. 2004, Becker et al. 2007, Loyola et al. 2007), climate change (Pounds et al. 2006, Blaustein and Johnson 2003), introduction of exotic species (Kats and Ferrer 2003) and diseases (Lips et al. 2003).

Despite Brazil showing the highest worldwide diversity of amphibians (Segalla et al. 2012), it does not rank within the first positions in the number of endangered species. This is probably due to the lack of data regarding most Brazilian species, once the country is also the world leader in species classified as Data Deficient (DD) – near 25% of the assessed species (IUCN 2008). Many of this species could be facing extinction but are not in the scope of conservation actions, since we cannot determine the major threats to their populations due to the absence of adequate sampling along the Brazilian territory (Trindade-Filho et al. 2012, Verdade et al. 2012, Campos et al. 2013, Morais et al. 2013).

Brazil’s Atlantic Forest is one of the five most important global hotspots of biodiversity (Myers et al. 2000). It harbors more than half of the country’s amphibians (Haddad et al. 2013) and exhibits high endemism rates (> 85% of species) for this taxonomic group (Cruz and Feio 2007). Such high biodiversity is faced with the current situation of the biome, with fewer than 11.7% of its original area remaining and only 1% of its total area being legally protected (Ribeiro et al. 2009). In addition, the number of amphibian species is expected to decline within the Atlantic Forest network of protected areas due to climate change, according to projections made by Lemes et al. (2014).

Given the devastation of the Atlantic Forest biome, where more than half of the Brazilian amphibians live, coupled with the lack of information to assess the conservation status of many species, primary studies are urgently needed to overcome these shortfalls.

The central region of the Atlantic Forest – including the south of Bahia – was deemed as a zone of climatic stability during the Quaternary glaciations and was the greatest refuge for amphibians in the Atlantic Forest during that period (Carnaval et al. 2009). This region is regarded as an important center of diversification and endemism for plants (Thomas et al. 1998) and different groups of animals (Haffer 1974, Brown 1991, Bencke et al. 2006).

In a comparison of the diversity of trees from different tropical forests in the world, a forest remnant of southern Bahia was amongst those with the greatest richness and was thereby considered a hotpoint within the Atlantic Forest hotspot (Martini et al. 2007). The identification of areas with large numbers of endemic species and species diversity within the major global hotspots assists in planning conservation actions aimed at smaller areas that can be more easily managed and protected than larger ones (Murray-Smith et al. 2008).

The study of the species richness and species composition in a given area is essential to know the functional structure of biological communities, as well as to understand the dynamics between fragments, serving as an instrument that contributes in decisions relative to species conservation (Droege et al. 1998, Haddad 1998). As highlighted by Verdade et al. (2012), further investments in basic research – in particular regarding the collection of field data during inventories of fauna and taxonomic reviews – are necessary to better understand the extraordinary biodiversity of Brazilian amphibians as to properly assess their conservation status and help design conservation strategies for this taxonomic group.

There is a huge lack of information about the occurrence of amphibians in the State of Bahia, Brazil. This can be noticed by the frequent publication of notes regarding the increased distribution of species in the state (e.g. Orrico 2010, Dias et al. 2010, Camurugi et al. 2010, Dias et al. 2011) and the large number of new species described over the last years (e.g. Napoli et al. 2011a, Lourenço-de-Moraes et al. 2012, Teixeira-Jr et al. 2013, Caramaschi et al. 2013). Furthermore, only eleven scientific articles contain lists of amphibian species in the state (Silvano and Pimenta 2003, Juncá 2005, Juncá 2006, Bastazini et al. 2007, Protázio et al. 2009, Valdujo et al. 2009, Camurugi et al. 2010, Xavier and Napoli 2011, Valdujo et al. 2011, Garda et al. 2013, Lantyer-Silva et al. 2013). Most of the available information regarding amphibians of southern Bahia are results of the work of Silvano and Pimenta (2003). Despite the fact that a small sampling effort was applied (about four nights at each fragment), a considerable number of fragments were sampled in 19 localities and a total of 92 species were found.

Due to difficult access or the lack of suitability for agricultural purposes, most forest remnants that exist outside protected areas in southern Bahia are situated on slopes or mountain tops (Thomas et al. 1998, 2008). From the scientific point of view, though, these places are still little explored even considering areas of utmost biological importance (Martinelli 2007). As for the montane areas, the few existing data exhibit a high diversity of species (Amorim et al. 2005, 2009, Matos et al. 2010, Rocha and Amorim 2012), many of which endemic and new to science (e.g. Amorim and Leme 2009, Napoli et al. 2011a, Machado et al. 2013, Teixeira-Jr et al. 2013).

Accordingly, the objective of this study was to inventory the amphibians of the Private Reserve of Natural Heritage (RPPN) Serra Bonita, a montane area covered by Tropical Rainforest in southern Bahia State, Brazil. Our data reveal that Serra Bonita is one of the amphibian habitats with the greatest diversity in the world, a hotpoint within the Atlantic Forest hotspot for this taxonomic group.

## Materials and methods

### Study area

**Figure 1.**
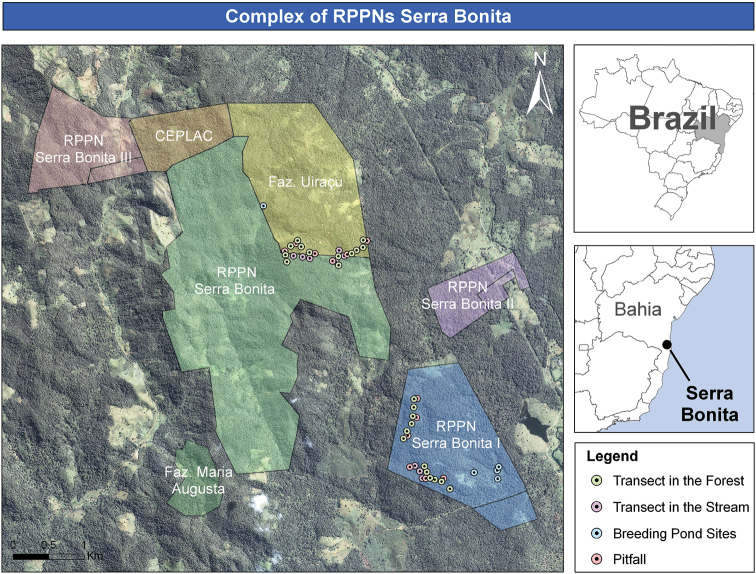
Map and sampling sites in the RPPN Serra Bonita, Bahia State, Northeastern Brazil.

The Serra Bonita Reserve (Figure 1) is located in the municipalities of Camacan and Pau-Brasil, Bahia State, Brazil (15°23'S, 39°33'W). The region is known as the “South Coast” and is located about 130 km from the city of Ilhéus and 526 km from the state capital – Salvador. Under a free lease agreement, the Uiraçu Institute administers around 20 km² from which 12 were transformed into a Private Reserve of Natural Heritage (PRNP or RPPN) located within the Central Corridor of the Atlantic Forest (Instituto Uiraçu 2009).

The area comprises steep altitudinal gradients (200–950 m a.s.l.) that cause changes in humidity and temperature along the gradient. The vegetation consists of a mosaic of forest fragments in different stages of ecological succession, with some *cabrucas* (cacao plantations where native woody plants are used for shading) and pastures. Preliminary floristic studies have identified 628 angiosperm species divided into 103 families (Amorim et al. 2009), and 173 species of ferns, as well as nine lycophytes; 44 of all species were new records for the State of Bahia and northeastern Brazil (Matos et al. 2010).

**Figure 2.**
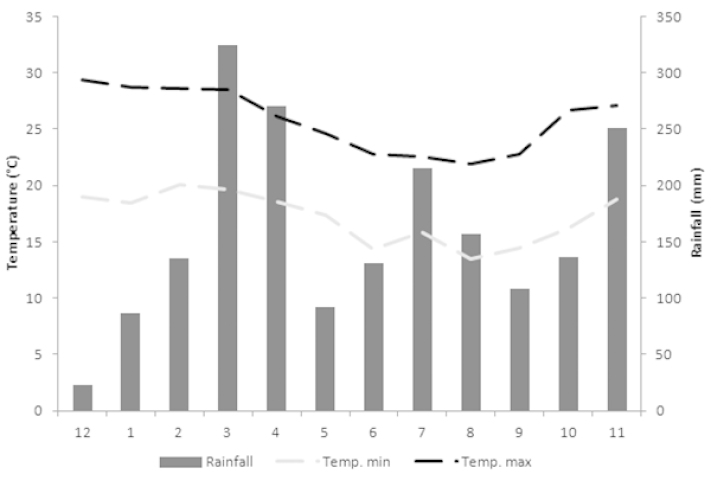
Rainfall data and minimum and maximum temperatures at the RPPN Serra Bonita between December 2009 and November 2010.

The climate in the region is the Af type of Köppen (1936) and is characterized as hot and humid without a dry season. During the study period, the average monthly rainfall was 160.9 mm (23.2–270.8 mm), while the average minimum air temperature was 17.2 °C (13.5–20.1 °C) and the maximum 25.8 °C (21.9–29.4 °C). Summarized data is displayed in Figure 2. These data were collected using two rain gauges installed at the site, one in the lower area (200 m a.s.l.) and another in the higher area (850 m a.s.l.), plus eight minimum and maximum thermometers placed along the forest, four in the low area and four situated upland.

### Collection and analysis of data

We conducted monthly field trips over six consecutive days from December 2009 to November 2010 and also recorded species during four other trips that lasted four days each, in July and December 2008, and February and May 2009, totaling 88 field days.

The inventory of amphibians was carried out using the following methods: (1) active visual and acoustic search (Heyer et al. 1994) via transects installed in the inner forest and streams; (2) pitfall traps (Cechin and Martins 2000); (3) active search in permanent and temporary ponds (Heyer et al. 1994); (4) opportunistic records done while the team moved along the trails, including specimens found by others.

From December 2009 to November 2010 we sampled 24 100-m transects placed in the inner forest and nine 50-m transects placed in streams. Active search was more frequently conducted by two researchers and occasionally by three. All transects were once inspected in every sampling month. Transects placed in the inner forest were traversed for 40 minutes, on average, totaling 192 hours/man. The transects placed in the streams, in turn, were traversed for 30 minutes, on average, totaling 54 hours/man.

We installed 12 pitfall traps, which were formed by four 100-L buckets shaped as “Y” and fitted with a one-meter-high canvas drift fence that connected them with five meter in length; the buckets remained open three nights per month over the year, totaling a sampling effort of 1728 buckets/day.

Five pools were occasionally sampled: two in the *cabruca*, another two in the pastures and another at the edge of the forest, with an overall sampling effort of about 25 hours. Temporary ponds formed on the used roads after heavy rain were also sampled.

The species that were found during reproductive activity were classified as having short (1–2 months), medium (3–7 months) and long (8–12 months) mating seasons. We considered the presence of males vocalizing as indicative of reproductive activity for species.

In order to verify a possible correlation between the monthly observed species richness and abiotic factors (rainfall, maximum and minimum temperatures), we used the Pearson’s correlation coefficient. Normality was checked with the Shapiro-Wilk test. Correlations were computed using the SPSS 13.0 software. To calculate the number of species that could occur within the RPPN Serra Bonita, we used the shapes relative to the area of occurrence of amphibians provided by IUCN (2008) overlapped with the limits of the two municipalities in which the PRNP is located (Pau Brasil and Camacan), using the ArcGIS program.

All animals were collected under license provided by IBAMA and/or the Chico Mendes Institute for Biodiversity Conservation upon permission of the directors of local reserves. Vouchers were deposited at the Museu da Universidade Estadual de Santa Cruz (MZUESC), Ilhéus, Bahia, Brazil (Appendix I).

## Results

**Figure 3.**
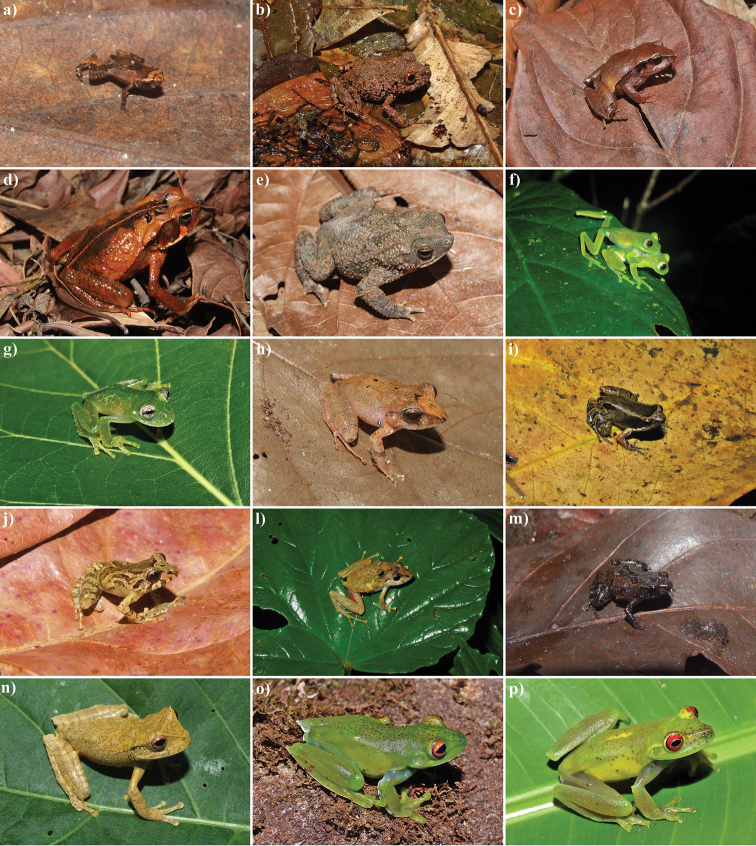
Anurans from the RPPN Serra Bonita, Bahia State, Northeastern Brazil. **a** *Brachycephalus pulex* **b** *Ischnocnema verrucosa* **c** *Ischnocnema* sp. 1 (gr. *parva*) **d** *Rhinella crucifer* **e** *Rhinella granulosa* **f** *Vitreorana eurygnatha* **g** *Vitreorana uranoscopa* **h** *Haddadus binotatus* **i** “*Eleutherodactylus*” *bilineatus* **j** *Pristimantis* sp. 1 **l** *Pristimantis vinhai* **m** *Adelophryne* sp. **n** *Gastrotheca pulchra* **o** *Aplastodiscus ibirapitanga*; and **p** Aplastodiscus cf. weygoldti. Photos by I. R. Dias.

**Figure 4.**
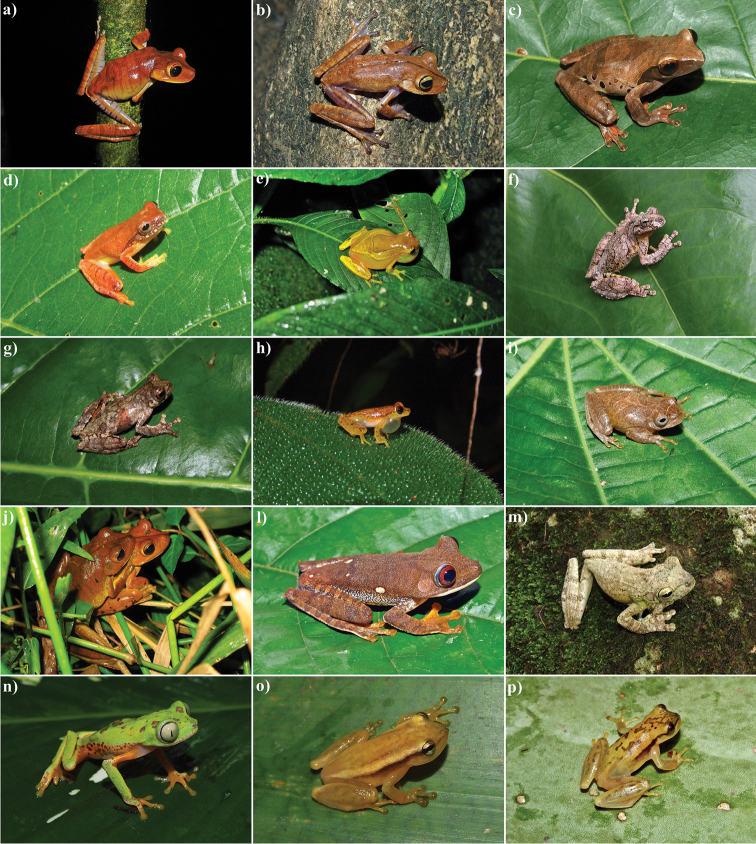
Anurans from the RPPN Serra Bonita, Bahia State, Northeastern Brazil. **a** *Bokermannohyla circumdata* **b** *Bokermannohyla lucianae* **c** *Dendropsophus anceps* **d** *Dendropsophus bipunctatus* **e** *Dendropsophus elegans* **f** *Dendropsophus novaisi* **g** *Dendropsophus giesleri* **h** *Dendropsophus haddadi* **i** *Dendropsophus minutus* **j** *Hypsiboas faber* **l** *Hypsiboas semilineatus* **m** *Itapotihyla langsdorffii* **n** *Phasmahyla spectabilis* **o** *Phyllodytes wuchereri* and **p** *Phyllodytes* sp. 1. Photos by I. R. Dias.

**Figure 5.**
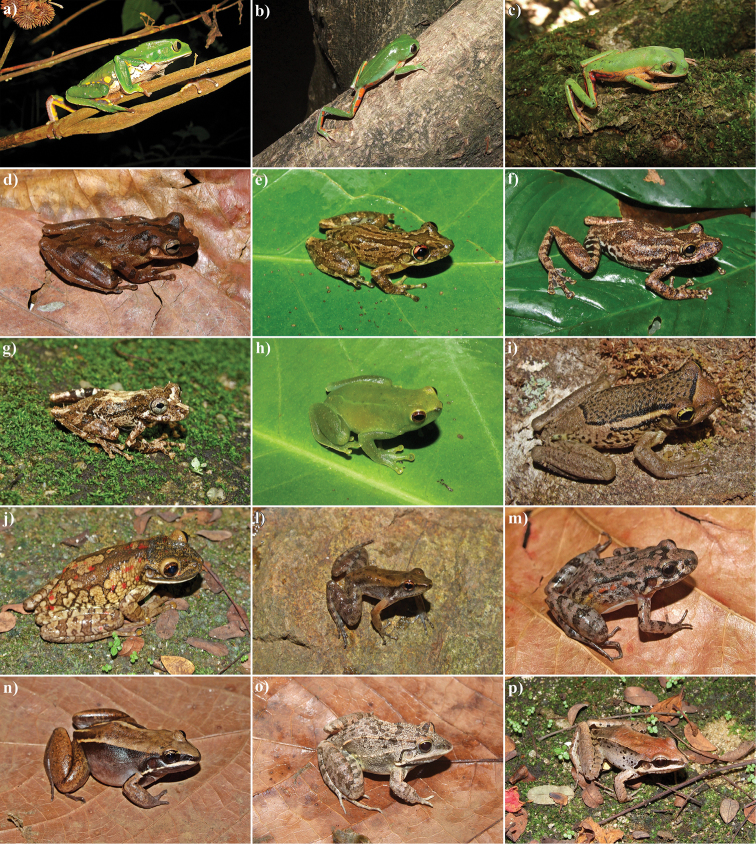
Anurans from the RPPN Serra Bonita, Bahia State, Northeastern Brazil. (a) *Phyllomedusa burmeisteri* **b** *Phyllomedusa nordestina* **c** *Phyllomedusa rohdei* **d** *Scinax eurydice* **e** *Scinax* sp. 1 **f** *Scinax* sp. 2 (gr. *rostratus*) **g** *Scinax strigilatus* **h** *Sphaenorhynchus prasinus* **i** *Trachycephalus mesophaeus* **j** *Trachycephalus nigromaculatus* **l** *Crossodactylus* sp. **m** Adenomera cf. thomei **n** *Leptodactylus cupreus* **o** *Leptodactylus fuscus* and **p** *Leptodactylus mystaceus*. Photos by I. R. Dias.

**Figure 6.**
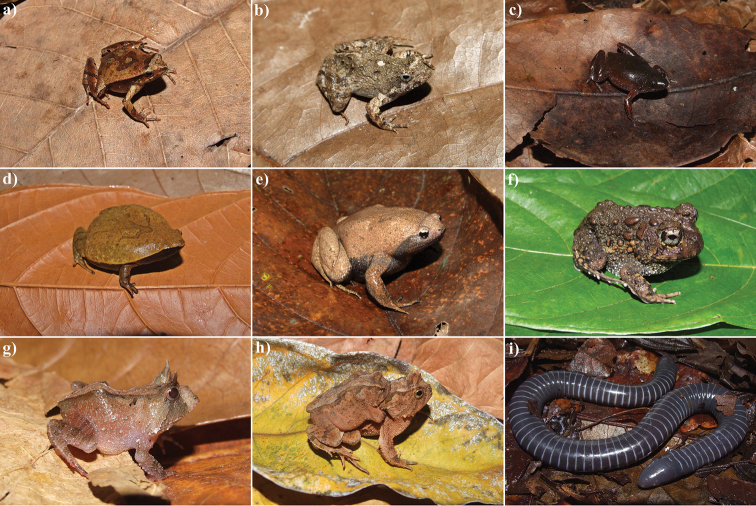
Anurans from the RPPN Serra Bonita, Bahia State, Northeastern Brazil. **a** *Physalaemus camacan* **b** *Physalaemus erikae* **c** *Chiasmocleis crucis* **d** *Stereocyclops histrio* **e** *Stereocyclops incrassatus* **f** *Odontophrynus carvalhoi* **g** *Proceratophrys renalis* **h** *Proceratophrys schirchi* and **i** *Siphonops annulatus*. Photos by I. R. Dias.

Eighty amphibian species were found in the RPPN Serra Bonita: a single species of Gymnophiona (*Siphonops annulatus* – Siphonopidae) and 79 species of anurans, allocated into 14 families (Table 1; Figures 3–6). The species richness observed in the Serra Bonita mountain is the second highest recorded for the Atlantic Forest biome and almost doubles those found in other locations in the State of Bahia (Table 2). It is noteworthy that only the Ecological Reserve of Michelin had a similar sampling effort as the one applied to our study in the state of Bahia.

**Table 1.** Amphibian species found in the RPPN Serra Bonita, southern Bahia, Brazil. Caption. IUCN (International Union for Conservation of Nature and Natural Resources): DD = Deficient Data; LC = Least Concern; VU = Vulnerable; NT = Near Threatened. Habitat: LL = Leaf litter or understory; S = Streams; P = ponds; B = bromeliads or epiphytes; Mating Activity = Period of mating activity: S = short (1–2 months); M = medium (3–7 months); and L = long (8–12 months); Sampling Method: OE = Opportunistic encounters; TF = Transect in the forest; TS = Transect in the streams; P = Pitfall; BP = Breeding pond sites. * = species only found in the inner forests; † = only acoustic record; # only recorded once or twice during the sampling.

Family/Species	IUCN	Habitat	Mating Activity	Sampling Method
AROMOBATIDAE				
*Allobates olfersioides* (Lutz, 1925)*	VU	LL e S	-	P, OE
BRACHYCEPHALIDAE				
*Brachycephalus pulex* Napoli, Caramaschi, Cruz & Dias, 2011*	-	LL	-	TF
*Ischnocnema verrucosa* Reinhardt & Lütken, 1862*	DD	S, LL	S	TS, TF, P
*Ischnocnema* sp. (gr. *parva*)*	-	LL	S	TS, TF, P, OE
BUFONIDAE				
*Rhinella crucifer* (Wied-Neuwied, 1821)	LC	P, LL	M	TF, TS, P, OE, BP
*Rhinella granulosa* (Spix, 1824)	LC	P, LL	S	OE, BP
*Rhinella hoogmoedi* Caramaschi & Pombal, 2006	LC	P, S	S	TF, TS, P, OE
*Rhinella jimi* (Stevaux, 2002)	LC	LL	-	OE
CENTROLENIDAE				
*Vitreorana eurygnatha* (A. Lutz, 1925)*	LC	S	L	TS
*Vitreorana uranoscopa* (Müller, 1924)*	LC	S	S	TS
CERATOPHRYIDAE				
*Ceratophrys aurita* (Raddi, 1823) #	LC	LL	-	OE
CRAUGASTORIDAE				
*Haddadus binotatus* (Spix, 1824)	LC	LL	L	TF, TS, P, OE
“*Eleutherodactylus*” *bilineatus* (Bokermann, 1975)*	LC	LL	S	TF, P, OE
*Pristimantis paulodutrai* (Bokermann, 1975)	LC	LL	-	OE
*Pristimantis* sp. 1*	-	B	L	TF, TS, OE
*Pristimantis* sp. 2*	-	LL	L	TF, TS, OE
*Pristimantis* sp. 3* #	-	LL	S	OE
*Pristimantis vinhai* (Bokermann, 1975)	LC	LL	L	TF, TS, OE
ELEUTHERODACTYLIDAE				
*Adelophryne mucronatus* Lourenço-de-Moraes, Solé & Toledo, 2012*	-	LL	-	TF, TS, OE
*Adelophryne* sp.*	-	LL	-	TF, TS, OE
HEMIPHRACTIDAE				
*Gastrotheca* sp.* #	-	B	-	TF, OE
*Gastrotheca pulchra* Caramaschi & Rodrigues, 2007*	DD	B	-	TF, TS
HYLIDAE				
Aplastodiscus cf. weygoldti (Cruz & Peixoto, 1985)*	NT	S	L	TF, TS, OE
*Aplastodiscus ibirapitanga* (Cruz, Pimenta & Silvano, 2003)*	LC	S	M	TF, TS, OE, BP
*Bokermannohyla circumdata* (Cope, 1871)*	LC	S	M	TF, TS, OE
*Bokermannohyla lucianae* (Napoli & Pimenta, 2003)*	DD	S	L	TF, TS, OE
*Dendropsophus anceps* (Lutz, 1929)	LC	P	M	TF, BP, OE
*Dendropsophus bipunctatus* (Spix, 1824) #	LC	P	S	BP
*Dendropsophus branneri* (Cochran, 1948)	LC	P	L	BP
*Dendropsophus elegans* (Wied-Neuwied, 1824)	LC	P	L	BP, TS
*Dendropsophus giesleri* (Mertens, 1950)	LC	P	M	BP
*Dendropsophus haddadi* (Bastos & Pombal, 1996)	LC	P	L	TS, BP
*Dendropsophus novaisi* (Bokermann, 1968) #	DD	P	S	BP, TF
*Dendropsophus minutus* (Peters, 1872)	LC	P	S	BP
Dendropsophus aff. oliveirai (Bokermann, 1963)	LC	P	L	BP
*Hypsiboas albomarginatus* (Spix, 1824)	LC	P	L	BP
*Hypsiboas atlanticus* (Caramaschi & Velosa, 1996) †	LC	P	M	BP, OE
*Hypsiboas crepitans* (Wied-Neuwied, 1824)	LC	P	M	TF, BP
*Hypsiboas exastis* (Caramaschi & Rodrigues, 2003) #	DD	P	S	OE
*Hypsiboas faber* (Wied-Neuwied, 1821)	LC	P	M	TF, TS, BP, OE
*Hypsiboas pombali* (Caramaschi, Pimenta & Feio, 2004)	LC	P	L	TF, TS, OE, BP
*Hypsiboas semilineatus* (Spix, 1824)	LC	P	S	BP
*Itapotihyla langsdorffii* (Duméril & Bibron, 1841) #	LC	P	S	BP
*Phasmahyla spectabilis* Cruz, Feio & Nascimento, 2008 *	DD	S	L	TF, TS
Phyllodytes cf. maculosus Peixoto & Cruz, 1988 †	LC	B	L	TF, TS, OE
*Phyllodytes melanomystax* Caramaschi, Da Silva & Britto-Pereira, 1992†	LC	B	L	TF, TS, OE
*Phyllodytes wuchereri* (Peters, 1873)	DD	B	L	TF, TS, OE
*Phyllodytes* sp. 1	-	B	L	TF, TS, OE
*Phyllodytes* sp. 2†	-	B	L	TF, TS, OE
*Phyllodytes* sp. 3†	-	B	L	TF, TS, OE
*Phyllodytes* sp. 4†	-	B	L	TF, TS, OE
*Phyllomedusa burmeisteri* Boulenger, 1882	LC	P	L	TF, TS, OE
*Phyllomedusa nordestina* Caramaschi, 2006	DD	P	M	BP
*Phyllomedusa rohdei* Mertens, 1926	LC	P	M	BP
*Scinax argyreornatus* (Miranda-Ribeiro, 1926)	LC	P	S	BP, OE
*Scinax juncae* Nunes & Pombal, 2010	-	P	M	BP
*Scinax x-signatus* (Spix, 1824)	LC	P	S	BP
*Scinax eurydice* (Bokermann, 1968)	LC	P	-	TF, TS, BP, OE
*Scinax* sp. 1 #	-	P	S	BP
*Scinax* sp. 2 (gr. *rostratus*) #	-	-	-	BP
*Scinax strigilatus* (Spix, 1824)*	DD	S	S	TF, TS, OE
*Sphaenorhynchus prasinus* Bokermann, 1973 #	LC	P	S	BP
*Trachycephalus mesophaeus* (Hensel, 1867)	LC	P	S	TF, BP, OE
*Trachycephalus nigromaculatus* Tschudi, 1838* #	LC	-	-	TF, OE
HYLODIDAE				
*Crossodactylus* sp.*	-	S	-	TS, OE
LEPTODACTYLIDAE				
Adenomera cf. thomei Almeida & Angulo, 2006*	LC	-	-	TS, P
*Leptodactylus cupreus* Caramaschi, Feio & São-Pedro, 2008 * #	DD	P	-	BP
*Leptodactylus fuscus* (Schneider, 1799)	LC	P	M	BP, OE
Leptodactylus cf. latrans (Steffen, 1815)	LC	P	M	TS, BP, OE, P
*Leptodactylus mystaceus* (Spix, 1824)	LC	-	-	OE
*Physalaemus camacan* Pimenta, Cruz & Silvano, 2005	DD	P	M	P, OE, BP
*Physalaemus erikae* Cruz & Pimenta, 2004	LC	P	M	TF, P, OE, BP
MICROHYLIDAE				
*Chiasmocleis crucis* Caramaschi & Pimenta, 2003	DD	P	S	TF, P, BP
*Stereocyclops histrio* (Carvalho, 1954)* #	DD	P	S	BP
*Stereocyclops incrassatus* Cope, 1870	LC	P	S	P, OE, BP
ODONTOPHRYNIDAE				
*Odontophrynus carvalhoi* Savage & Cei, 1965* #	LC	S	S	OE
*Proceratophrys renalis* (Miranda-Ribeiro, 1920)* #	-	LL	-	OE
*Proceratophrys schirchi* (Miranda-Ribeiro, 1937)*	LC	S, LL	L	TF, TS, P, OE
PIPIDAE				
*Pipa carvalhoi* (Miranda-Ribeiro, 1937) #	LC	P	-	BP
SIPHONOPIDAE				
*Siphonops annulatus* (Mikan, 1820)*	LC	LL	-	TF, P, OE

**Table 2.** Number of anuran species found in areas of greatest richness within the Atlantic forest of Brazil and the state of Bahia (abbreviations for Brazilian states as follows: BA: Bahia; ES: Espírito Santo; SP: São Paulo; RJ: Rio de Janeiro). For studies providing the sampling effort in days and the total duration of the fieldwork we provided both.

Localities	N	Time Sampled	Area (km²)	References
**ATLANTIC RAIN FOREST**				
Municipality of Santa Teresa (ES)	92	> 10 years	711	Almeida et al. 2011; Rödder et al. 2007
**RPPN Serra Bonita (BA)**	**80**	**88 days (16 months)**	**20**	**This study**
Reserva Biológica de Paranapiacaba (SP)	69	*	3.36	Verdade et al. 2009
Municipality of Rio de Janeiro (RJ)	68	> 5 years	1356	Izecksohn and Carvalho-e-Silva 2001
Estação Biológica da Boracéia (SP)	67	> 5 years	164.5	Heyer et al. 1990
Parque Estadual Carlos Botelho (SP)	65	76 days (1 year)	377.9	Forlani et al. 2010
Parque Estadual Turístico do Alto Ribeira (SP)	60	15 days (3 months)	357.1	Araujo et al. 2010
**STATE OF BAHIA**				
Serra da Jibóia and Serra do Timbó – Elísio Medrado and Amargosa	53	#	~ 100	Juncá 2006; Camardelli and Napoli 2012
Reserva Ecológica da Michelin – Ituberá	48	52 days (13 months)	9.75	Camurugi et al. 2010
Parque Estadual da Serra do Conduru – Ilhéus, Uruçuca and Itacaré	45	13 days (2 months)	92.7	Pimenta 2005
Reserva Sapiranga – Mata de São João	37–40	36 days (8 months)	6	Juncá 2006; Bastazini et al. 2007
RPPN Veracel – Porto Seguro	39	4 days	80.9	Silvano and Pimenta 2003
Fazenda Vista Bela – Guaratinga	34	4 days	4.65	Silvano and Pimenta 2003
Projeto de Assentamento Zumbi dos Palmares – Camamu	32	4 days	18.3	Silvano and Pimenta 2003

* Mainly scientific data collections;

# richness estimated based on the literature and scientific data collections (Camardelli and Napoli 2012) and 12 days (4 months) of fieldworks (Juncá 2006).

Among the species found in the study area that were already classified by the IUCN (n = 63), the vast majority (n = 48; 76.1%) are considered as “Least Concern” and 20.6% (n = 13) as data deficient to assess their conservation status (Table 1). Two species deserve special attention: Aplastodiscus cf. weygoldti and *Allobates olfersioides*, respectively, are currently considered near threatened and vulnerable to extinction by the IUCN (2013). A recent scientific assessment of the extinction risk of the Brazilian fauna concluded that *Bokermannohyla lucianae* and *Phasmahyla spectabilis* can be considered as near-threatened with extinction (Subirá et al. 2012).

Amongst the species recorded during mating activity (n = 61), 23 exhibited prolonged mating (37.7%) and the same number (n = 23) was observed to mate on few occasions during the study period, whereas 24.5% showed an intermediate mating period (Table 1). Thirty-five of the species observed during mating activity were found in temporary or permanent ponds, twelve in streams, six in plant litter, and eight species were seen vocalizing in bromeliads and epiphytes (Table 1).

The highest variety of species was recorded through opportunistic encounters (50 species). The search at breeding sites resulted in the registration of an intermediary of sampled species (38 species), however, with a high number of exclusive species (18 species), even using a smaller effort hour/man. Data on efficacy of the methodologies used can be checked in Table 3.

**Table 3.** Number total and exclusive species sampled by each sampling method with the respective sampling effort.

Sampling Method	Richness	Exclusive	Sampling Effort
Opportunistic encounters	50	7	-
Transect in the Forest	40	1	192 (hours/man)
Breeding pond sites	38	18	25 (hours/man)
Transect in the streams	36	2	54 (hours/man)
Pitfall	15	0	1728 (buckets/day)

A total of 16 species (20%) was only recorded once or twice during the sampling (Table 1). Most of these species are explosive breeder and were sampled after heavy rainfall at the study area.

There was a significant positive correlation between richness and monthly rainfall (r = 0.65, DF = 10, p = 0.027), but the same was not observed for the minimum (r = -0.22, DF = 10, p = 0.49) and the maximum (r = -0.31, DF = 10, p = 0.33) temperatures.

## Discussion

Brazil harbors 946 amphibian species (Segalla et al. 2012) and near 543 of them have been recorded in the Atlantic Forest (Haddad et al. 2013). The State of Bahia is home to about 190 amphibian species. The amphibian community of RPPN Serra Bonita includes almost 15% of the overall species recorded in the Atlantic Forest and more than 40% of the species of Bahia State. Of the 20 anuran families that occur in Brazil (Frost 2014) only six (most of them Amazonian species) have not been sampled at the Serra Bonita mountain (Allophrynidae, Alsodidae, Ceuthomantidae, Cycloramphidae, Dendrobatidae and Ranidae). These are expressive numbers, since such high diversity was detected concentrated in the 20 km² area forming the RPPN Serra Bonita.

The place known to show the largest amphibian richness in the Atlantic Forest is the municipality of Santa Teresa, Espírito Santo State, with 92 species (Rödder et al. 2007, Almeida et al. 2011). Yet, this amphibian diversity was obtained considering the entire municipality of Santa Teresa (711 km²), with a sampling effort of more than ten years, including records from different scientific collections. Thus, the amphibian diversity obtained in the RPPN Serra Bonita deserves special attention, as it comprises a smaller sampling area and effort. Even thus, the observed species richness is the second-highest ever recorded in a single study in the Atlantic Forest.

Most areas with high amphibian diversity in the Atlantic Forest are located in southeastern Brazil and are usually associated with mountainous locations (Heyer et al. 1990, Izecksohn and Carvalho-e-Silva 2001, Verdade et al. 2009, Forlani et al. 2010, Araujo et al. 2010). Aside from being the region where amphibians were better studied in Brazil, it concentrates the largest number of research groups working on amphibians (Rossa-Feres et al. 2011, Campos et al. in press).

Three out of the 80 species recorded at RPPN Serra Bonita (*Eleutherodactylus* “*bilineatus*”, *Pristimantis paulodutrai*, *Pristimantis vinhai*) are endemic to the State of Bahia (Juncá and Pimenta 2004, Peixoto and Pimenta 2004a, Pimenta and Juncá 2004) and another six (*Adelophryne mucronatus*, *Brachycephalus pulex*, *Chiasmocleis crucis*, *Physalaemus camacan*, *Physalaemus erikae*, *Stereocyclops histrio* and *Scinax strigilatus*) are endemic to southern Bahia State (Cruz and Pimenta 2004, Pimenta et al. 2005b, Pimenta et al. 2007, Targino and Wild 2009, Napoli et al. 2011a, Lourenço-de-Moraes et al. 2012, Forlani et al. 2013). *Phyllodytes wuchereri* and *Dendropsophus novaisi* are species known from less than three localities (Caramaschi et al. 2004, Peixoto and Pimenta 2004b), whereas *Bokermannohyla lucianae* was only known from the municipality of Una (Dias et al. 2011). Thus, 15% of the species are endemic to the Bahia State and considering the species identified at specific level or as “cf.”, 77.2% are endemic to the Atlantic Forest biome according to Haddad et al. (2013). Out of the 80 species recorded at the site, 29 were only recorded in the inner forests.

In this study we expand the geographic distribution of *Vitreorana uranoscopa* (Figure 3G) from Santa Teresa municipality, Espírito Santo State (Rödder et al. 2007) to the RPPN Serra Bonita (near 530 km north). Furthermore, two new species whose genera were only known to occur northward up to the Santa Teresa municipality were recently described from the State of Bahia. *Crossodactylodes septentrionalis* from Serra das Lontras (about 20 km from Serra Bonita in the municipality of Arataca) (Teixeira-Jr et al. 2013) and *Brachycephalus pulex* from RPPN Serra Bonita (Napoli et al. 2011a) both species being endemic to their type localities. To date, the two areas (municipality of Santa Teresa and RPPN Serra Bonita) have 33 species in common, but this number could even be larger. In addition, some species with northernmost distribution known to Santa Teresa have been found in the state of Bahia (Freitas et al. 2004, Orrico 2010, Camurugi et al. 2010).

Much of the shared amphibians between the two areas are composed of typical lowland species or species associated to mountainous regions but with a wide distribution range. The Santa Teresa region is located within the northern range of the Serra da Mantiqueira. The montane areas of southern Bahia do not belong to this mountain chain. The presence of restricted endemisms in both regions, especially species living at higher altitudes, may indicate that historic geomorphological and climatic factors may have contributed to turn these mountain ranges into key areas of speciation, as has been suggested for the northern range of the Serra da Mantiqueira (Napoli 2005, Napoli et al. 2011b). However, molecular studies with phylogeographic approaches are needed to allow a better understanding of the role of the bahian mountain ranges in the diversification of the local anuran fauna.

Amongst the 80 species found in the area, 18 were cited without a specific name or were classified as similar or described in groups of species (see Table 1). Some of these belong to groups that are taxonomically complex and difficult to identify (e.g. *Adelophryne* spp.; *Crossodactylus* spp.; *Scinax* spp.). The increase in research and scientific advances involving the specimens collected during this study can reveal the existence of new species or identify the presence of species to date unknown to exist in the State of Bahia. A recent analysis of samples collected during the development of this inventory resulted in the description of *Brachycephalus pulex* (Napoli et al. 2011a). Moreover, new species may be described for the region.

The sampling effort of our study was focused on transects installed within streams and inner forest fragments. Thus, some habitats and environments were not intensively sampled, such as the *cabrucas*, permanent and temporary ponds, as well as some areas of the RPPN complex that were not logistically feasible to be sampled. These areas had ponds and swamps in the inner forest, a feature that was not found in the areas sampled in our study. Future standardized effort including these environments might further enhance the richness of amphibians in the area.

In tropical regions, richness and mating activity are largely influenced by rainfall (Aichinger 1987, Duellman and Trueb 1994). Studies conducted in areas with seasonality detected a positive correlation between the sampled richness, rainfall and air temperature (Toledo et al. 2003, Santos et al. 2007, Kopp et al. 2010). Much of the amphibian community of Serra Bonita exhibited prolonged and intermediate mating activity (62% of spp.). According to Crump (1974) this is the pattern expected for non-seasonal tropical regions. As the area does not present a marked seasonality, most species meet suitable conditions allowing them to mate during much of the year.

In this study we detected a positive correlation between the sampled species richness and the monthly rainfall. In addition, it is likely that there was an increase in the number of species sampled during the months with higher rainfall indexes due to the appearance of explosive breeding species (e.g. *Dendropsophus novaisi*, *Stereocyclops incrassatus*, *Chiasmocleis crucis*, *Itapotihyla langsdorffii*, *Hypsiboas exastis*, *Scinax* sp. 1 and *Stereocyclops histrio*).

## Final remarks

When comparing the amphibian species richness of RPPN Serra Bonita with that found at other sampled sites in the Bahia State, we notice a large disparity, since most locations exhibit less than half the number of species recorded in this study. This can be associated to the structural complexity of the sampled environment, which involves changes in temperature, rainfall, humidity and plant coverage along the altitudinal gradient in the study area, but also to the lack of systematic studies in most localities in the state of Bahia that still have large forest fragments and a mosaic of different ecosystems and biomes that need to be further explored from the scientific standpoint.

The largest Late Pleistocene refugium for amphibians has been estimated in southern Bahia and northern Espírito Santo states (Carnaval et al. 2009). During the quaternary glaciations, this zone of climatic stability probably allowed the coexistence and the diversification of a greater number of species in Serra Bonita, as compared to other places. Accordingly, the encounter of areas with a high concentration of amphibian species in this region was not unexpected. Serra Bonita may have been the first area to reveal such astonishing species richness, but other locations within the refugium have the same potential to support a high diversity of amphibians.

According to the IUCN (2008), the boundaries of the two municipalities of the studied RPPN (Camacan and Pau Brasil), are located within the area of geographical distribution of 26 other species (*Aparasphenodon brunoi*, *Chiasmocleis carvalhoi*, *Chiasmocleis schubarti*, *Cycloramphus fuliginosus*, *Dendropsophus decipiens*, *Dendropsophus nanus*, *Hypsiboas albopunctatus*, *Leptodactylus mystacinus*, *Leptodactylus natalensis*, *Leptodactylus spixi*, *Leptodactylus viridis*, *Macrogenioglottus alipioi*, *Phyllodytes luteolus*, *Physalaemus crombiei*, *Physalaemus cuvieri*, *Physalaemus marmoratus*, *Physalaemus signifer*, *Pleurodema diplolister*, *Proceratophrys laticeps*, *Pseudopaludicola mystacalis*, *Scinax alter*, *Scinax fuscomarginatus*, *Scinax fuscovarius*, *Sphaenorhynchus palustris*, *Sphaenorhynchus pauloalvini* and *Thoropa miliaris*). These species have not been found during our field activities, but may potentially occur in the region. Most of them are typically encountered in lowland areas. Despite being a mountain with well preserved forests on its top several smaller forest fragments and abandoned cacao plantations still remain in the lowland areas of the RPPN, offering potential habitat for theses species. Two recently described species (*Crossodactylodes septentrionalis* and *Dendrophryniscus oreites*) from Serra das Lontras (ca. 20 km from Serra Bonita) and the species *Gastrotheca recava* and *Gastrotheca megacephala* (Teixeira-Jr et al. 2012, Teixeira-Jr et al. 2013, Recoder et al. 2010) possibly also may occur in the study area. Thus, we estimate that Serra Bonita may be the home for 100–110 amphibian species, representing one of the largest diversities for the group in the world.

The number of research institutes with graduate programs and professionals working on biodiversity in the State of Bahia has steadily increased during the last decade. The amount of information is expected to significantly increase in the coming years. Nonetheless, if we consider the vast extent of the territorial borders of Bahia, this increase in physical and human resources should be encouraged and further increased in order that we can better understand the diversity of anuran fauna in the State of Bahia. A further step is to encourage funding agencies to sponsor scientific expeditions and medium and long term studies in the State – mostly basic studies – such as inventories of the local fauna.

Serra Bonita has a total area of 7500 hectares. One goal of the Uiraçu Institute is to protect about half that area. To date, about 2000 ha are under protection of the institute, which manages and protects the areas under a free lease agreement. Coupled with the wide diversity and endemism of amphibians found in the area, the finding of new species and new records for Bahia State make Serra Bonita a hotpoint for amphibians within the Atlantic Forest hotspot and, consequently, a place for the implementation of priority conservation measures aiming the increase of the protected area.

## Appendix I

**Specimens examined**

Voucher specimens of the present study were deposited in the Museu de Zoologia da Universidade Estadual de Santa Cruz (MZUESC) at Universidade Estadual de Santa Cruz, municipality of Ilhéus, Bahia State, Brazil.

AROMOBATIDAE

*Allobates olfersioides* – MZUESC 8305.

BRACHYCEPHALIDAE

*Brachycephalus pulex* – MZUESC 8352, 8604-05, 10338-40.

*Ischnocnema verrucosa* – MZUESC 8323, 8464, 8829, 8993, 9090.

*Ischnocnema* sp. (gr. *parva*) – MZUESC 8057, 8059, 8234, 8312-13, 8366-68, 8460-61.

BUFONIDAE

*Rhinella crucifer* – MZUESC 8428-29, 8310.

*Rhinella granulosa* – MZUESC 9016.

*Rhinella hoogmoedi* – MZUESC 8380, 8601, 8904.

CENTROLENIDAE

*Vitreorana eurygnatha* – MZUESC 8308, 8373-75, 8441-42, 8520, 8911.

*Vitreorana uranoscopa* – MZUESC 8630, 9000-01.

CRAUGASTORIDAE

*Haddadus binotatus* – MZUESC 8045, 8327-28, 8421, 8435, 8437, 8611, 8906, 8909, 8927.

“*Eleutherodactylus*” *bilineatus* – MZUESC 8359, 8457, 8616-17, 8999.

*Pristimantis* sp. 1 – MZUESC 8058, 8133, 8370-72, 8530, 8619-20, 8841-42, 9084-85.

*Pristimantis* sp. 2 – MZUESC 13272-73.

*Pristimantis vinhai* – MZUESC 8115, 8332, 8338, 8382, 8536-37, 8542, 8608, 8621, 8629.

ELEUTHERODACTYLIDAE

*Adelophryne mucronatus* – MZUESC 8458, 8068.

*Adelophryne* sp. – MZUESC 8049, 8358, 8447, 8838-40.

HEMIPHRACTIDAE

*Gastrotheca* sp. – MZUESC 8827.

*Gastrotheca pulchra* – MZUESC 8120, 8314, 8463, 8828.

HYLIDAE

Aplastodiscus cf. weygoldti – MZUESC 8139, 8443, 8531-32, 8532, 8572, 8612-13, 8830-34.

*Aplastodiscus ibirapitanga* – MZUESC 8307, 8318, 8369, 8571, 8598-99, 8600, 8994, 9086.

*Bokermannohyla circumdata* – MZUESC 8360-61, 8439-40, 8526-29, 8618, 8826, 9074-76.

*Bokermannohyla lucianae* – MZUESC 8295-97, 8995.

*Dendropsophus anceps* – MZUESC 8419, 8857, 9048-49.

*Dendropsophus bipunctatus* – MZUESC 10332-34.

*Dendropsophus branneri* – MZUESC 8474-75.

*Dendropsophus elegans* – MZUESC 8849-50.

*Dendropsophus giesleri* – MZUESC 8590-92, 8855, 10335.

*Dendropsophus haddadi* – MZUESC 8362, 8476, 8568-69, 8577-82, 9087.

*Dendropsophus novaisi* – MZUESC 8565, 9025-27.

*Dendropsophus minutus* – MZUESC 8853, 9005.

Dendropsophus aff. oliveirai – MZUESC 8477-78, 8566-67.

*Hypsiboas albomarginatus* – MZUESC 8471, 8854, 8903.

*Hypsiboas crepitans* – MZUESC 8899.

*Hypsiboas faber* – MZUESC 8326, 8379.

*Hypsiboas pombali* – MZUESC 8302, 8378, 8631, 9018.

*Hypsiboas semilineatus* – MZUESC 13268-71.

*Itapotihyla langsdorffii* – MZUESC 9023-24.

*Phasmahyla spectabilis* – MZUESC 8150-51, 8294, 8298, 8303.

*Phyllodytes wuchereri* – MZUESC 8134, 8319, 9052.

*Phyllodytes* sp. 1 – MZUESC 8135-36, 8574.

*Phyllomedusa burmeisteri* – MZUESC 8309, 8470.

*Phyllomedusa nordestina* – MZUESC 9003-04.

*Phyllomedusa rohdei* – MZUESC 8846, 9015.

*Scinax argyreornatus* – MZUESC 8473, 8588-89.

*Scinax juncae* – MZUESC 8856, 13056-13068

*Scinax x-signatus* – MZUESC 8573, 8593-96.

*Scinax eurydice* – MZUESC 8041, 8113, 8432, 8467, 8603, 8847-48.

*Scinax* sp. 1 – MZUESC 8570, 8584-87.

*Scinax* sp. 2 (gr. *rostratus*) – MZUESC 8573.

*Scinax strigilatus* – MZUESC 8365, 8623-24, 8626, 8996, 9053, 9081, 9083.

*Sphaenorhynchus prasinus* – MZUESC 8597.

*Trachycephalus mesophaeus* – MZUESC 8315, 9019.

*Trachycephalus nigromaculatus* – MZUESC 8299, 8905.

HYLODIDAE

*Crossodactylus* sp. – MZUESC 8056, 8121-22, 8521.

LEPTODACTYLIDAE

Adenomera cf. thomei – MZUESC 8138, 8317, 8364.

*Leptodactylus cupreus* – MZUESC 9041.

*Leptodactylus fuscus* – MZUESC 9002, 9017.

Leptodactylus cf. latrans – MZUESC 8915.

*Leptodactylus mystaceus* – MZUESC 8040.

*Physalaemus camacan* – MZUESC 9045-47.

*Physalaemus erikae* – MZUESC 8423, 9006, 9042-44.

MICROHYLIDAE

*Chiasmocleis crucis* – MZUESC 9028–29, 9031–34, 9036.

*Stereocyclops histrio* – MZUESC 9037-40.

*Stereocyclops incrassatus* – MZUESC 8422, 9020-22.

ODONTOPHRYNIDAE

*Odontophrynus carvalhoi* – MZUESC 8564.

*Proceratophrys renalis* – MZUESC 10341.

*Proceratophrys schirchi* – MZUESC 8152, 8300, 8468-69, 8602, 10342-45.

PIPIDAE

*Pipa carvalhoi* – MZUESC 7360.

SIPHONOPIDAE

*Siphonops annulatus* – MZUESC 8376, 8459, 8913, 9056, 9070.
